# Evaluation of Urine Reagent Strip as a Tool for Routine Diagnosis of Maternal Urogenital Schistosomiasis at Antenatal Clinic Visit in Munyenge, South West Region, Cameroon

**DOI:** 10.1155/2019/2972630

**Published:** 2019-12-06

**Authors:** Godlove Bunda Wepnje, Judith Kuoh Anchang-Kimbi, Leopold Gustave Lehman, Helen Kuokuo Kimbi

**Affiliations:** ^1^Department of Zoology and Animal Physiology, Faculty of Science, University of Buea, P.O. Box 63, Buea, Cameroon; ^2^Department of Animal Biology, Faculty of Science, University of Douala, P.O. Box 24157, Douala, Cameroon; ^3^Department of Medical Laboratory Sciences, Faculty of Health Sciences, The University of Bamenda, P.O. Box 39, Bambili, Cameroon

## Abstract

Urine reagent strip used in detecting microhaematuria has been recommended in pregnancy for diagnosis of urogenital schistosomiasis (UGS) during routine antenatal care (ANC). This study evaluated its sensitivity, specificity, and predictive values in the diagnosis of maternal UGS using filtration method as a reference test. We also assessed the variation in its performance in the diagnosis of UGS using multiple-sample collection. A total of 93 pregnant women reporting for first ANC clinic visit at any of the three functional health care centres (Munyenge Integrated Health Centre, Banga Annex Health Centre, and Trans African Health Centre) were enrolled and followed up for three consecutive monthly visits. Urine samples were observed microscopically for *S*. *haematobium* egg using urine filtration and screened for microhaematuria and proteinuria using urine reagent strips. Twenty-two (23.7%) out of the 93 women were diagnosed for UGS, all of whom showed *S*. *haematobium* egg excretion during all three visits. There was a significant difference (*p* < 0.001) between the prevalence of *S*. *haematobium* infection and the prevalence of microhaematuria. The intensity of infection was significantly higher in microhaematuria-positive women compared with microhaematuria-negative cases. Sensitivity of reagent strip ranged from 54.5 to 59.1%, while specificity was above 98.0% (range: 98.6–100%). The measure of agreement between urine filtration and reagent strip method was substantial (0.61–0.8) irrespective of different sampling periods. Urine reagent strip is a moderately sensitive method in the detection of UGS and will most likely identify women with high egg load burden. Proper diagnosis of schistosomiasis during pregnancy is recommended.

## 1. Introduction

Maternal schistosomiasis is a public health concern in endemic countries of sub-Saharan Africa where successful and sustainable control programmes are lacking [1–5]. It is estimated that approximately 10 million women annually have the infection during pregnancy [6] where maternal urogenital schistosomiasis (UGS) is associated with serious adverse effects on pregnancy such as preterm labour, low birth weight, and maternal anaemia [3, 4, 7]. Besides pregnancy associated morbidities, female genital involvement associated with *S*. *haematobium* infection has been reported as a plausible risk factor for HIV acquisition [8]. Praziquantel is widely acknowledged as the most important, rapid, and cost-effective method of reducing morbidity due to schistosome infections among risk groups [9]. Management of infected pregnant women through treatment with praziquantel is recommended by WHO, but many endemic countries are reluctant to adopt this policy because of the lack of sufficient safety data [6, 10]. However, findings from controlled trial studies continue to support the expansion treatment policies to include pregnant women [10, 11]. Studies in Cameroon have shown that the use of pipe-borne water eliminates infection [5], but it is not sustainable as pregnant women in endemic areas still make contact with water through domestic and bathing activities [4, 5].

*Schistosoma haematobium* infection can be diagnosed by several approaches. The urine filtration technique using polycarbonate filter membranes is recommended as the gold standard for *S*. *haematobium* egg detection [12]. Although this method costs 0.1–0.3 US dollars (USD) per test [13], its use in resource-constrained settings is limited due to unavailability. Thus, low-cost methods such as single-ply paper towels as urine filters have been improvised to substitute for the polycarbonate filters in resource limited settings [14]. Rapid detection of UGS in infected individuals is necessary for prompt intervention through treatment. Rapid immunodiagnostic kit (lateral flow urine-CCA dipstick) for diagnosis of schistosomiasis is commercially available, but poor sensitivity for *S*. *haematobium* [15, 16] has been reported. Several studies have validated the use of urine reagent strip for the detection of microhaematuria, a proxy indicator for *S*. *haematobium*. Compared with proteinuria and leukocyturia, microhaematuria is the most widely studied indicator in endemic areas with high sensitivity to *S*. *haematobium* infection [17–20]. Besides, urine reagent strips are used to aid in quick mapping surveys in endemic regions as it is rapid, easy to use, and less time consuming [21, 22].

Microhaematuria may be a useful rapid diagnostic tool for schistosomiasis in pregnant women and has been recommended to be introduced in routine antenatal care in *Schistosoma*-endemic areas [19]. To assess the performance characteristics of urine reagent strip in diagnosis of maternal UGS, we evaluated its sensitivity, specificity, and predictive values using filtration method as a reference diagnostic test. Furthermore, we assessed the variation in the performance of reagent strip test in the diagnosis of UGS in pregnant women followed up for three consecutive monthly visits.

## 2. Materials and Methods

### 2.1. Study Area

The study was conducted in Munyenge, South West Region, Cameroon. As previously reported, Munyenge is mesoendemic for *S*. *haematobium* infection [23]. This work was a prospective cohort study and part of a larger epidemiological study on maternal urogenital schistosomiasis conducted between November 2016 and January 2018 [5].

### 2.2. Study Population and Design

Pregnant women reporting for the first ANC clinic visit at any of the three functional health care centres (Munyenge Integrated Health Centre, Banga Annex Health Centre, and Trans African Health Centre) were enrolled and followed up for three consecutive monthly visits.

 
*Inclusion criteria*. Pregnant women who had lived in the area for at least six months and gave written informed consent were included in the study.
 
*Exclusion criteria*. Nonregular participants and visitors were not eligible.

#### 2.2.1. Sample Size Determination

The sample size for the study was calculated using the formula *n* = *Z*_*α*/2_^2^ Se (1 − Se)/*d*^2^ × Prev [24] where *n* = sample size, *Z*_*α*/2_ = 1.96: confidence level test statistic at the desired level of significance, Se = 98.8%: sensitivity of previous published data [25], Prev = 46.8%: prevalence of maternal UGS [4], and *d* = 0.05: acceptable error willing to be committed. The minimum sample size was estimated as *n* = 39. The sample size was then increased by 40% to a minimum of 93 participants who were followed up for three ANC visits.

### 2.3. Rapid Diagnostic Test and Parasitological Examination

Collection and examination of urine samples were done at the laboratory unit of the health facility. During the monthly visits, enrolled study participants were provided with dry, clean, and screw capped 20 mL universal bottles and instructed to collect midstream urine between 10 am and 2 pm. Urine reagent strips (Mission®, ACON Laboratories, Inc., USA) were used to screen for microhaematuria and proteinuria. To ensure valid results, strips were stored under required temperature (2°C to 30°C). Each lot of reagent strip was checked for quality control by comparing with known negative and positive controls. The urinalysis was performed according to the manufacturer's instructions.

The presence and number of *S*. *haematobium* eggs were detected under a microscope using the urine filtration method with polycarbonate filters (Sterlitech, Kent, WA, United States of America) [26]. Briefly, 10 ml of urine was filtered through a nylon filter and examined microscopically for schistosome eggs. Eggs were graded into light infection (1–49 eggs/10 ml urine) and heavy infection (≥50 eggs/10 ml urine) [27]. Any negative slide was confirmed by a second microscopist.

#### 2.3.1. Outcome Variables

- Microhaematuria-positive was defined as a urine sample that had a trace or positive reagent strip colour reaction (+, ++, +++).
- Proteinuria-positive was defined as a urine sample that had a trace or positive reagent strip colour reaction (+, ++, +++).
- *S*. *haematobium*-positive was defined as a urine filtration slide that contained at least one *S*. *haematobium* egg.

### 2.4. Statistical Analysis

Data collected was validated and analysed using SPSS version 20.0 (SPSS Inc., Chicago, USA). Data was grouped into first, second, and third ANC visit. The statistical tests performed included Pearson's chi-square for comparison of proportions and the Mann–Whitney and Kruskal–Wallis tests to determine significant differences in the *S*. *haematobium* egg intensity. Diagnostic performance of urine reagent strip was determined by calculating sensitivity, specificity, positive and negative predictive values, and accuracy. Using the urine filtration method as the gold standard, sensitivity of urine reagent strip was calculated as the proportion of positives that were correctly identified when compared with the standard test. The specificity of the test was calculated as the proportion of negatives that were correctly identified when compared with the diagnostic reference test. Positive predictive value was the probability that a disease is present when the test is positive while negative predictive value was the probability that the disease is not present when the test is negative. Accuracy was the overall probability that a patient will be correctly classified. *p* values <0.05 were considered statistically significant.

The agreement between the urine filtration versus reagent strip was determined using kappa (*κ*)-statistics. The *κ*-statistics were interpreted as follows: <0.0, 0.0–0.2, 0.21–0.4, 0.41–0.6, 0.61–0.8, 0.81–0.99, and 1.0, indicating no agreement, slight agreement, fair agreement, moderate agreement, substantial agreement, almost perfect agreement, and perfect agreement, respectively [28].

### 2.5. Ethics Approval and Consent to Participate

Ethical clearance (No2017/0481/UB/FHS/IRB) was obtained from the University of Buea, Faculty of Health Sciences Institutional Review Board, and administrative authorisation from the South West Regional Delegation of Public Health, Buea and District Medical Officer for Muyuka Subdivision. Written and verbal informed consent was obtained before enrolment into the study. Participation was voluntary and study participants were assured of confidentiality and anonymity of data.

## 3. Results

A total of 93 pregnant women were enrolled and followed up for three months. The mean age of pregnant women was 23 ± 4.9 years (range: 15–38). The distribution of sociodemographic and stream behaviour characteristics of study participants is presented in Table 1. Generally, women made fewer visits (<3 times/week) to the stream. Nonetheless, women who reported stream contact carried out one or two stream activities (domestic or bathing). Most women had no access to piped-borne water (Table 1).

Pregnant women were screened for *S*. *haematobium* infection, microhaematuria, and proteinuria for three consecutive monthly visits. The prevalence of infection due to *S*. *haematobium* and morbidity indicators did not vary between visits (Table 2). There was a significant difference (*p* < 0.001) between *S*. *haematobium* infection and microhaematuria. However, no variation was seen across the different visits. No association was recorded between infection status and proteinuria except for the first visit which showed marginal significance (*p*=0.049) (Table 3). The intensity of infection (egg counts) was significantly higher in microhaematuria-positive women compared with microhaematuria-negative cases. The association was similar across the visits (Table 3).

The sensitivity (detection of *S*. *haematobium*-positive cases) and specificity (detection of negative cases) of microhaematuria and proteinuria in the diagnosis of *S*. *haematobium* infection are shown in Table 4. Screening for microhaematuria alone enabled accurate detection of more than 50% (range: 54.5–59.1) of *S*. *haematobium*-positive cases and above 98.0% (range: 98.6–100) of negative cases. PPV was above 92.0% (range: 92.3–100) (Table 3). The sensitivity of proteinuria was consistently low (0–13.6%) in detecting *S*. *haematobium*-positive cases. The measure of agreement between urine filtration method and the urine reagent strip was substantial (0.61–0.8) across visits.

## 4. Discussion

This study evaluated the performance characteristics of urine reagent strip in the diagnosis of maternal UGS at routine antenatal care. Furthermore, we assessed the variation in its performance using multiple urine test samples at different collection periods.

Our study clearly showed that microhaematuria was significantly associated with maternal urogenital schistosomiasis which suggests that it is a good biomarker for infection in pregnant women in endemic areas. This is in line with reports by other authors [19, 29–31]. *Schistosoma haematobium*-associated microhaematuria results from bladder lesions that result from the deposition of the parasite's spiny eggs in the submucosa [30]. Inconclusive evidence has suggested *S*. *haematobium*-associated glomeruli pathology leading to proteinuria [17, 19, 32, 33]. Contrarily, our study did not show association between the occurrence of proteinuria and *S*. *haematobium* infection among pregnant women. However, proteinuria has other causes that are unrelated to *S*. *haematobium* infection which include genitourinary infection and/or the sequelae of genital mutilation [33] and preeclampsia [19]. Hence, a suspected UGS-induced proteinuria by urine reagent strip in pregnant women must be confirmed by microscopy. The association between egg load and microhaematuria has been reported [25, 34] with high intensity of *S*. *haematobium* egg excretion significantly associated with higher odds to present with microhaematuria [22]. Our findings support this association as women who shed eggs with detectable microhaematuria had significantly high egg load excretion.

The sensitivity of microhaematuria (range: 54.5–59.1%) to detect infected individuals in our study was similar to that (61.1%) reported in a larger survey in the same study area [5]. Similarly, moderate sensitivity of microhaematuria has been reported in other parts of *Schistosoma*-endemic regions of Africa [22, 35] although some studies have recorded higher sensitivity values (range: 82–98%) [19, 20, 25]. Knopp et al. [22] suggested that high intensity of infection may influence accuracy of reagent strip results. Likewise, the association between microhaematuria and egg intensity observed in the present study suggests that the reagent strip method may correctly identify cases with high egg load burden.

PPV was very high among pregnant women indicating that a greater fraction of microhaematuria in such setting is related to schistosomiasis. NPV and PPV are well known to be prevalence dependent; the higher the prevalence of the disease in a given setting, the higher the PPV and the lower the NPV [36, 37]. In this respect, our findings on the NPV and PPV imply that a positive reagent strip test would necessarily relate to a positive *S*. *haematobium* infection. Comparing the results of urine reagent strip and filtration, we found that the agreement between both methods was substantial. Consequently, reagent strips, often used to monitor medical conditions of pregnant women during antenatal care, could become a very useful tool for quick diagnosis of schistosomiasis for rational control measure.

It is generally recommended that testing for schistosomiasis be repeated by follow-up with two or more consecutive visits for increased accuracy [38]. In this present study, we demonstrated stability in egg excretion among pregnant women irrespective of the different sampling periods.

## 5. Conclusion

Urine reagent strip is a moderately sensitive method in the detection of maternal UGS. Sampling at different time periods did not influence diagnostic performance of urine reagent strip. Routine urinalysis will most likely identify women with high infection burden; thus, proper diagnosis and management of schistosomiasis during pregnancy is recommended to forestall *S*. *haematobium*-induced adverse pregnancy outcomes and reduce transmission.

## Figures and Tables

**Table 1:** Characteristics of study participants.

Variable	Category	Number examined (*N*)	%
Age group (years)	≤20	36	38.7
	21–25	34	36.6
	>25	23	24.7

Marital status	Single	34	36.6
	Married	59	63.4

Level of education	At least primary	25	26.9
	At least secondary	68	73.1

Occupation	Housewife	9	9.7
	Business	28	30.1
	Student	33	35.5
	Farmer	23	24.7

Stream usage	Yes	71	76.3
	No	22	23.7

Stream activity	Domestic contact + bathing	18	25.4
	Domestic contact only	53	74.6

Stream visits per week	<3 times	47	66.2
	≥3 times	24	33.8

Access to piped-borne water	Yes	23	24.7
	No	70	75.3

**Table 2:** Prevalence of *S*. *haematobium* infection, microhaematuria, proteinuria, infection intensity, and mean egg load/10 ml of urine stratified by ANC visits.

Outcome	Category	1^st^ ANC visit % (*n*)	2^nd^ ANC visit % (*n*)	3^rd^ ANC visit % (*n*)	*χ*2; *p* value
*S*. *haematobium* infection status	Positive	23.7 (22)	23.7 (22)	23.7 (22)	0.00; 1.0
	Negative	33.3 (71)	33.3 (71)	33.3 (71)	

Microhaematuria	Positive	14.0 (13)	14.0 (13)	15.1 (14)	0.06; 0.971
	Negative	86.0 (80)	86.0 (80)	84.9 (79)	

Proteinuria	Positive	5.4 (5)	1.1 (1)	4.3 (4)	2.70; 0.260
	Negative	94.6 (88)	98.9 (92)	95.7 (89)	

Infection intensity	High	27.3 (6)	18.2 (4)	22.7 (5)	0.52; 0.772
	Low	72.7 (16)	81.8 (18)	77.3 (17)	

Mean (±SD) egg load/10 ml of urine		38.5 (52.6)	28.3 (33.1)	27.0 (26.6)	^*∗*^1.93; 0.147

^*∗*^ = Kruskal–Wallis test (H).

**Table 3:** Association between *S*. *haematobium* egg infection status and infection intensity, proteinuria, and microhaematuria.

Association between *S*. *haematobium* infection status, microhaematuria, and proteinuria						
ANC visit	Indicators		Number	*S*. *haematobium* infection status		^#^ *p* value
				Pos % (*n*)	Neg % (*n*)	

1^st^ visit	Microhaematuia	Pos	13	92.3 (12)	7.7 (1)	<0.001
		Neg	80	12.5 (10)	87.5 (70)	
	Proteinuria	Pos	5	60.0 (3)	40.0 (2)	0.049
		Neg	88	21.6 (19)	78.4 (69)	
2^nd^ visit	Microhaematuria	Pos	13	100.0 (13)	0.0 (0)	<0.001
		Neg	80	11.2 (9)	88.8 (71)	
	Proteinuria	Pos	1	0.0 (0)	100.0 (1)	0.576
		Neg	92	23.9 (22)	76.1 (70)	
3^rd^ visit	Microhaematuria	Pos	14	92.9 (13)	7.1 (1)	<0.001
		Neg	79	11.4 (9)	88.6 (70)	
	Proteinuria	Pos	4	0.0 (0)	100.0 (4)	0.255
		Neg	89	24.7 (22)	75.3 (67)	

Association between infection intensity and microhaematuria						
ANC visit	Status	*N*		Mean (±SD) egg load/10 ml urine (range)		^&^ *p* value
1^st^ visit	Eggs/microhaematuria	12		61.2 (62.1)		0.004
	Eggs/no microhaematuria	10		11.4 (15.7)		
2^nd^ visit	Eggs/microhaematuria	13		41.6 (37.6)		<0.001
	Eggs/no microhaematuria	9		9.1 (7.3)		
3^rd^ visit	Eggs/microhaematuria	13		38.3 (29.4)		0.017
	Eggs/no microhaematuria	9		10.7 (7.4)		

^#^ = Chi-square test, ^&^ = Mann–Whitney *U* test, Pos = positive, Neg = negative.

**Table 4:** Performance of microhaematuria and proteinuria in the diagnosis of UGS among pregnant women.

ANC visit	Diagnostic parameters	Microhaematuria (95% CI)	Proteinuria (95% CI)
1^st^ visit	Sensitivity	54.5 (34.6–73.1)	13.6 (4.7–33.3)
	Specificity	98.6 (92.4–99.7)	97.2 (90.3–99.2)
	PPV	92.3 (66.7–98.6)	60.0 (23.1–88.2)
	NPV	87.5 (78.5–93.1)	78.4 (68.7–85.7)
	Accuracy	88.2 (80.0–93.3)	77.4 (67.9–84.7)
	*κ* value	0.6	0.1

2^nd^ visit	Sensitivity	59.1 (38.7–76.7)	0.0 (—)
	Specificity	100.0 (94.9–100.0)	98.6 (92.4–99.7)
	PPV	100.0 (77.2–100.0)	0.0 (—)
	NPV	88.7 (80.0–94.0)	76.1 (66.4–83.6)
	Accuracy	90.3 (82.6–94.8)	75.3 (65.6–82.6)
	*κ* value	0.7	−0.02

3^rd^ visit	Sensitivity	59.1 (38.7–76.7)	0.0 (—)
	Specificity	98.6 (92.4–99.7)	94.4 (86.4–97.8)
	PPV	92.9 (68.5–98.7)	0.0 (—)
	NPV	88.6 (79.7–93.9)	75.3 (65.4–83.1)
	Accuracy	89.2 (81.3–94.0)	72.0 (62.2–88.1)
	*κ* value	0.7	−0.08

PPV = positive predictive value; NPV = negative predictive value.

## Data Availability

All datasets used to support the findings of this study are included within the article.

## Abbreviations

UGS: Urogenital schistosomiasis
ANC: Antenatal care clinic.
